# Lot quality survey: an appealing method for rapid evaluation of vaccine coverage in developing countries – experience in Turkey

**DOI:** 10.1186/1471-2458-8-240

**Published:** 2008-07-16

**Authors:** Banu Cakir, Sarp Uner, Fehminaz Temel, Levent Akin

**Affiliations:** 1 Hacettepe University, Faculty of Medicine, Department of Public Health, TR-06100 Ankara, Republic of Turkey

## Abstract

**Background:**

Vaccine-preventable diseases cause significant morbidity and mortality worldwide and in developing countries in particular. Information on coverage and reasons for non-vaccination is vital to enhance overall vaccination activities. Of the several survey techniques available for investigating vaccination coverage in a given setting, the Lot Quality Technique (LQT) remains appealing and could be used in developing countries by local health personnel of district or rural health authorities to evaluate their performance in vaccination and many other health-related programs. This study aimed to evaluate vaccination coverage using LQT in a selected semi-urban setting in Turkey.

**Methods:**

A LQT-based cross-sectional study was conducted in Kecioren District on a representative sample of residents aged 12–23 months in order to evaluate coverage for routine childhood vaccines, to identify health units with coverage below 75%, and to investigate reasons for non-vaccination.

**Results:**

Based on self-reports, coverage for BCG, diphtheria-pertussis-tetanus (DPT-3), oral polio-3, hepatitis-3, and measles vaccines ranged between 94–99%. Coverage for measles was below 75% in five lots. The relatively high educational and socioeconomic status of parents in the study group alone could not minimize the "considerable" risk of vaccine-preventable diseases in the District and dictates a continuity of efforts for improving vaccination rates, with special emphasis on measles. We believe that administrative methods should be backed up by household surveys to strengthen vaccination monitoring and that families should be trained and motivated to have their children fully vaccinated according to the recommended schedule and in a timely manner.

**Conclusion:**

This study identified vaccine coverage for seven routine vaccines completed before the age of 24 months as well as the areas requiring special attention in vaccination services. The LQT, years after its introduction to health-related research, remains an appealing technique for rapid evaluation of the extent of a variety of local health concerns in developing countries, in rural areas in particular, and is very efficient in determining performance of individual subunits in a given service area. Training of local health personnel on use of the LQT could expedite response to local health problems and could even motivate them in conducting their own surveys tailored to their professional interests.

## Background

Vaccine-preventable diseases cause significant morbidity and mortality worldwide and in developing countries in particular. Information on performance at the local level is vital to enhance overall vaccination coverage. Routine surveillance programs are essential for baseline estimations and proper follow-up of vaccination services, yet they are not always complete and reliable. Resources and manpower are usually limited, data are available only for those "who seek medical advice/service", and/or data collection forms may/may not include the information required. Periodic household surveys may be beneficial in reaching even those least likely to seek medical help, or who cannot access the services. Several survey techniques can be used for determining vaccination coverage in a given setting, though each has certain advantages and disadvantages given the resources [1-4].

The Lot Quality Technique (LQT) has long been used for evaluating vaccine coverage. It can easily be conducted by local staff/trained interviewers; is particularly efficient when the population has an overall high coverage, but subunits are heterogeneous in coverage; and is remarkably advantageous when coverage in individual subunits of the population needs to be evaluated [3,5,6]. Local health personnel of district or rural health authorities in developing countries could be easily trained in planning and conducting lot quality surveys to evaluate their performance in dealing with a variety of health-related issues in their service areas. Such practices will not only lead them to resolve local health concerns in a rapid and independent manner but will also save financial resources, manpower and expertise they may need to outsource.

An administrative protocol has recently been signed between our university and the Kecioren Health District in Ankara, founding the Kecioren Health Training and Research (HTR) District. The Kecioren HTR District endows the university with a site for field-based training of our graduate and undergraduate students, while faculty members provide educational and technical support to local health personnel in improving their services on an as-needed basis.

Based on the routine surveillance program of the Kecioren District Health Administration in 2006, the coverage for routine childhood vaccines was below acceptable levels in some areas, while it was "reportedly" exceeding 100% in some other areas. The considerable heterogeneity in coverage raised concerns on the validity and "completeness" of the surveillance data and on the possibility of a weakness in enumerating the "population at risk" in some districts.

Given that administrative data are not trustworthy in providing robust estimates for vaccination coverage, a population-based survey was conducted in Kecioren Health District. The study aimed to evaluate coverage for seven routine childhood vaccines, to identify health units with "unacceptably low" coverage (if any), and to investigate reasons for non-vaccination. This paper summarizes the findings of this survey, as an example of how LQT can be used as an appealing method for rapid evaluation of vaccine coverage in developing countries.

## Methods

**Figure 1:**
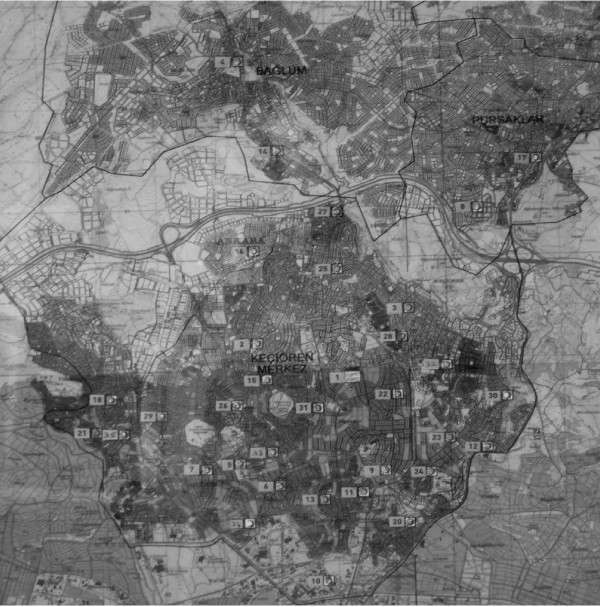
**The map of Keciören Health District, including 33 primary health care units**. Three health centers (number 34, 35, 36) have been built recently and were not included in this study.

A cross-sectional study was conducted in the Kecioren District of Ankara, Turkey, on a random sample of children aged 12–23 months, to establish valid and reliable estimates for evaluating the effectiveness of the routine vaccination services provided in the region and to ultimately provide scientific evidence for tailoring future interventions in the region to maximize vaccination coverage among children. The District Health Administration provides health services to a population of 750 000 residents, all living in urban settings, including 12 881 children aged 12–23 months (administrative data, 2006, unpublished). There are 33 primary health care units in the District that are obliged to periodically enumerate all residents (including children) in their service area, regardless of where they apply for health service, and to provide vaccination services to all children residents, free of charge. However, vaccination-related data on an individual basis may be incomplete, given that children may receive vaccination services from facilities other than these primary health care units, including six hospitals, 24 private outpatient clinics, and several private physician offices. Thus, the study collected data via a household survey, based on parents' (or caregivers') self-reports and vaccination cards, whenever available (Figure 1).

In this study, vaccine coverage was calculated for Kecioren District as a whole, and identified those health care settings with coverage below 75% as the units with "unacceptably low" coverage. Seven vaccines were studied. A child was considered as "fully-immunized" if s/he was vaccinated for BCG, and had received three doses of DPT (diphtheria, pertussis, tetanus), oral polio, and hepatitis B and at least one dose of measles vaccine (either monovalent measles or trivalent). The quality of services was also evaluated based on the two criteria of owning a vaccination card and "timeliness" of vaccinations.

The LQT was used in the study to allow rapid assessment of vaccination coverage in the District. It also served to further familiarize the local health staff with an easy, convenient and valuable technique that could be used to study a variety of other local health concerns and to obtain district-wide prevalence rates together with health unit-specific evaluations of their "performance".

The LQT was originally derived from production-line industry to categorize the quality of batches of a product as either permissible or unsatisfactory based on the production of defective units in each batch, found by inspecting a sample of unit "lots". In evaluating vaccination services in a selected area, the population is divided into individual (administrative or service) units, a sufficient number of subjects are randomly chosen from each unit, and vaccination status of these selected children is evaluated [5,6]. In this study, each primary health care unit service area (n = 33) is designated as a "lot", providing data for calculation of coverage and to determine the "quality" of services.

The *total sample size *required was determined based on the total population aged 12–23 months, the acceptable levels of vaccination coverage (i.e., the *upper *"desired" level for coverage and the *lower *"threshold" level that can be accepted for a given vaccine), and the desired *level of confidence *and *level of accuracy *in reaching a valid estimate [5,6]. In the study, the "acceptable range" was set as 75% to 95% (the national target); any lot with a coverage of <75% was identified as having "unacceptably low" coverage and thus requiring urgent attention. The coverage for each vaccine was calculated for the total population, with a confidence level of 95 ± 3% [5].

The required *total sample size *for the area was 1089, corresponding to a minimum of 33 children (*lot sample size*) from each lot. In the study, lot #10 had a total of only 26 children aged 12–23 months; thus, this lot's sample size was set at 26. Selection of children (*the sampling points*) was established randomly, prior to the field study, to minimize the risk of selection bias. At the HTR center, the map of each lot was examined; sampling points were scattered throughout the lot area, based on the number of streets in each lot. If the number of streets in a given lot was more than 33, 33 streets were selected randomly for each lot and one child was selected from each street; otherwise, the number of children from each street in the lot was determined as 33/number of streets in that lot. In the field, based on the total number of children to be selected from the houses situated on a (chosen) street, the interviewers randomly selected one house, checked whether an eligible child lived there and (if so) interviewed the parent/caregiver. House selection continued on the same street till an eligible age was found, and the caregiver was interviewed. In houses where two "eligible" children were present, only one was selected randomly for the interview, with the interviewer blinded to his/her vaccination status. The interviewers collected data from the entire sample determined for each lot and did not stop if the threshold of children was met in any lot, to allow estimation of district-wide coverage.

The decision value, i.e. the number of non-vaccinated children above which the lot was identified as having "unacceptably low" level of coverage, was 4 for all lots in the study except one (n = 3, lot #10). The decision value was identified using Sample LQ software package [7].

### 1. Data collection tool

Data were based on self-reports of the child's caregiver (mother, if possible). A standardized and pilot-tested questionnaire was used in face-to-face interviews in the household setting. A standard 24-item questionnaire (most items were close-ended, with multiple choices) was completed in each house in about 15 minutes. Data were collected on sociodemographic and vaccine-related characteristics of children, family characteristics, and reasons for non-vaccination (if applicable).

### 2. Statistical analysis

Analyses included frequency and percent distributions, calculation of coverage for individual vaccines for children in Kecioren District, and identification of lots with coverage below 75%. Based on LQT (5), all analyses were conducted respecting sampling weights, calculated as inverses of the relevant sampling fractions. Multivariable logistic regression modeling was used to identify significant predictors of "full immunization" (i.e., having all doses of all required seven vaccines versus having at least one dose of any vaccine missing). The final model presented is the most explanatory ("full") model, including all independent predictors of vaccination status and potential confounders as data permitted to study. Variable selection for the model was based on statistical significance (at alpha = 0.05) in bivariate and stratified analyses of our own data and/or was based on literature knowledge. All analyses were conducted using SPSS version 14 and the Complex Samples module of SPSS version 15 statistical software package (Chicago, IL).

### 3. Ethical issues

Written permission was obtained from the Provincial Health Directorate, Ankara, and verbal approval of the study participants (parent/caregiver) was obtained prior to data collection. In the original report provided to the involved parties, the names of the individual primary health care units (lots) were given so that further interventional activities could be planned appropriately. In this manuscript, on the other hand, each health care unit is represented with its lot number to respect the confidentiality of unit-specific performances.

## Results

Vaccination coverage for 12–23-month-old children residing in Kecioren District in February 2007 was calculated based on a representative sample of 1111 children from service areas of 33 primary health care centers [3 maternal and child health care and family planning (MCH-FP) centers and 30 health centers]. Of the 1111 children, 51.0% were males and 41.3% were the first child in the family. Vaccine-related information was obtained mainly from the mother (85.9%) or from other caregivers when the mother could not be reached (i.e., father, grandparent, or others for 5.5%, 4.8% and 3.8% of children, respectively). The mean (± standard deviation) ages of mothers and fathers were 28.7 ± 5.8 years and 32.5 ± 5.8 years, respectively. In the study population, 39.5% of the mothers and 55.8% of the fathers were high school or university graduates. Four out of five (82.3%) children had some sort of health insurance. One of the parents was currently employed in 96.7% of the cases, and fathers (95.6%) were more likely than mothers (13.2%) to be employed.

Forty percent of study participants had no siblings, whereas 36.3% had one and 23.6% had two or more siblings. Of the participants, 42.3% had at least one family member aged < 15 years, while 39.8% had two and 14.2% had three family members aged < 15 years. Only 16.1% of the children were living in slums.

Children were taken for vaccination mainly by their mothers (97.8%), fathers (17.5%), or other relatives (6.9%); more than one family member took this responsibility in some cases. The mostly preferred health care setting for vaccination was health centers (82.4%), followed in order of frequency by MCH-FP centers (8.7%), private outpatient clinics (5.8%), and private-(4.8%) or state-(4.1%) hospitals.

In Kecioren District, 98.8% of children (reportedly) have a vaccination card but only 57.5% (range: 11.3%–91.7% in different lots) of those parents/caregivers could present the card when asked in the interviews; 41.3% reportedly had a card, but could not show it when requested. Of the interviewed parents/caregivers, 1.0% reported that the child did not have a vaccination card. Of the card owners, 97.2% reported that they "always" carry their card with them when presenting at the vaccination site. For vaccination, 69.1% go to the health care center on foot, and only 23.0% require a private vehicle to reach vaccination services. Time to reach vaccination site was less than 5 minutes for 31.4% of the participants, and more than 30 minutes for only 1.8% of the participants.

Of the participants, 91.3% were fully vaccinated. BCG vaccination was not done in 0.6%. The prevalence of missing doses of DPT vaccines was 0.9% for one dose, 0.2% for two doses, and 0.4% for three doses. Similarly, the prevalence of missing doses of oral polio vaccine was 0.8%, 0.2%, and 0.7% for one, two, and three doses. With respect to hepatitis B vaccine, 2.2% of children were missing one dose, while 0.1% and 0.4% were missing two and three doses, respectively.

Based on self-reports, coverage for BCG vaccine is 99.4%. All children in the study were examined for their BCG scars, excluding children who were not at home at the time of interview and whose caregivers did not approve examination. The prevalence of presence of a BCG scar was 63.9% in all participants.

The Kecioren-wide coverage for three doses of DPT, oral polio, and hepatitis B was 98.5%, 99.4%, and 97.3%, respectively. Prevalence of having at least one dose of vaccination against measles was 93.9% (Table 1). These indicated that coverage for all routinely required childhood vaccines was above nationwide accepted levels except for measles, which was below the nationally desired level of 95%. Strikingly, coverage for measles vaccine was below 75% in five lots (lots 10, 18, 21, 27, 33); thus, performance of these primary health care units was considered as "unacceptably low", requiring immediate interventions.

**Table 1:** Distribution of coverage in 12–23-month-old children and time of vaccination among those with a vaccination card according to vaccine studied (Kecioren District, 2007)

	**Coverage ** **(%)**	**Vaccination age ** **was detected ** **(%) (n)§**	**Appropriately timed ** **vaccinations** (%)*	**Vaccination Month†**	
				**Mean ± standard deviation**	**Median (25%;75%)**
**BCG**	99.4 (63.9)	54.6 (n = 603)	87.2	2.35 ± 0.93	2.20 (2.20;2.47)
**DPT-3**	98.5	53.4 (n = 584)	67.0	5.08 ± 1.75	4.60 (4.30;5.33)
**OPV-3**	99.4	53.0 (n = 585)	63.9	5.06 ± 1.35	4.67 (4.33;5.47)
**Hepatitis B-3**	97.3	52.4 (n = 566)	79.2	8.97 ± 1.72	9.20 (7.80;9.73)
**Measles**	93.9‡	6.0 (n = 63)	77.9	9.43 ± 0.68	9.47 (9.10;9.80)
**MMR**		41.9 (n = 437)	76.3	12.85 ± 1.12	12.47 (12.30;12.93)

* Nationally recommended vaccination age is 2, 3, 4, 9, and 9/12 months for BCG, DPT-3, oral polio-3, hepatitis B-3 and measles/MMR vaccines, respectively. These months are regarded as "appropriately timed vaccinations" in the study. Percentages were calculated out of total number of children for whom vaccination date was available, as given in parentheses in the second column of the table.

**† **As obtained from vaccination card (if available).

‡ Child had at least one dose of either type of vaccine against measles (monovalent measles or MMR).

^§ ^Percentage gives the proportion of children who were reported as vaccinated for the given vaccine and for whom the vaccination date was detected at the time of the interview. Numbers in parentheses are the number of children for whom vaccination date was detected.

Depending on the type of vaccine, possibility of obtaining vaccination dates from vaccination cards (when available) ranged between 6.0% (measles) and 54.6% (BCG). Among these children for whom vaccination data was available, the "timeliness" of vaccination ranged between 77.3% (measles, mumps, rubella-MMR) and 87.2% (BCG). In the study, vaccination ages did not distribute normally; thus, median vaccination ages were calculated for each vaccine. These median ages were 2.2, 4.6, 4.7, 9.2, and 9.5/12.5 months for BCG, DPT-3, oral polio-3, hepatitis B-3, and measles/MMR vaccines, respectively (Table 1).

The study also aimed to identify significant predictors of "full immunization" in children residing in Kecioren District. In logistic regression modeling of "full immunization", the following covariates were studied simultaneously: source of information (other versus mother), gender of the child, age of the child at the time of the interview (months), mother's age (years), father's age (years), current occupational status of the parents (neither of the parents currently working versus at least one parent currently working), health insurance of the child (none versus any), being the first child of the family (yes versus no), educational attainment of the mother and father (primary school graduate or less, secondary school graduate, high school or university graduate), presence of a vaccination card (card information could not be provided or had no card versus had a card), and number of family members aged < 15 years. Among these, three variables were identified as significant predictors of a child's immunization status. Controlling for the other covariates, immunization status improved (i.e., child is fully immunized) as age increased (OR = 1.20, 95% C.I.= 1.12–1.28) (p < 0.001), whereas it significantly decreased if none of the parents was working (OR = 0.19, 95% C.I. = 0.07–0.50) (p = 0.002). Similarly, a negative association was detected between presence of vaccination card and "full immunization". Those having a vaccination card and who could show it when requested were three times less likely than their counterparts without cards to be vaccinated with all seven vaccines and all required doses (OR = 0.32, 95% C.I. = 0.18–0.60) (p = 0.001).

Lastly, reasons for non-vaccination were investigated when a child had missing vaccines and/or doses. Ninety-seven children (8.7%) were missing at least one dose of any of the vaccines studied, including those missing more than one vaccine. A variety of reasons were cited by the parents for non-vaccination, including: unaware of the vaccination site, fear of vaccine's side effects, misperceptions, familial reasons, no one available to take the child to the vaccination site, the child was sick and was not taken to the vaccination site, the child was sick and the health personnel did not approve vaccination, the vaccine was not available at the health care unit visited, the waiting period was too long, or the child was still young for the given vaccine, etc. In some situations, parents cited more than one reason, while a significant percent of the mothers/caregivers did not give a specific reason for non-vaccination. Table 2 summarizes the reasons as reported by all caregivers who did not complete their children's vaccination schedule.

**Table 2:** Distribution of the reasons for non-vaccination*§ (Kecioren District, 2007) (n = 96)

**Reasons Cited**	**BCG**	**DPT**	**OPV**	**Hepatitis B**	**Measles/MMR**
Did not know the site for vaccination	-	-	-	-	1
Scared of the side effects	2	2	2	2	2
Misperceptions (false believes)	2	1	1	1	2
Nobody available to take the child to the vaccination site	-	-	-	-	1
Familial problems	-	-	-	-	3
Child was sick, was not taken for vaccination	1	2	2	2	6
Child was sick, health personnel did not approve vaccination	-	-	-	-	1
Vaccine was not available	-	-	-	-	1
Waiting period was too long	1	-	-	-	-
Date for vaccination is yet to come	-	-	-	-	18
Other **†**	3	3	5	3	8
Not stated/not known	2	11	9	20	28
Total number of children missing at least one dose^§^	7	17	17	26	65

* Given the scarcity of the number of non-vaccinated children or those missing at least one dose of a vaccine, percentages were not calculated.

**† **Did not have an ID, did not have vaccination card, was out of town or was busy with some other responsibilities, forgot the date of vaccination.

§ Some mothers/other caregivers stated more than one reason for non-vaccination; thus, column totals do not necessarily equal the number of children missing at least one dose of vaccine.

## Discussion

The LQT has long been used in health for a variety of topics, with special prominence in vaccine-related research. In a recent review of literature from records of the World Health Organization, World Bank, Medline and five different electronic databases, out of 805 LQT-based health-related researches conducted between 1984 and 2004 by researchers and governmental and private institutions, 266 were on vaccine coverage [8].

The main reasons for common use of LQT in vaccine-related research are its ease in application, non-requirement of a sampling frame record and/or large population sizes, ability to determine performance of individual subunits in the population and to identify those with "unacceptably low" coverage, and the possibility of choosing a range of different confidence interval and accuracy levels. Technical ease of using LQT together with its efficiency makes this technique appealing in many developing countries, especially in rural areas where technical assistance may be less accessible and use of alternative techniques such as simple random sampling or cluster sampling would not be suitable for lack of household or individual lists or need for assistance in statistical analysis (e.g., to control for design effect or to use weighted analysis where sampling is disproportionate to size).

The World Health Organization has been providing technical support to many countries in assessing vaccine coverage following vaccination campaigns and helps in local capacity building in performing such studies. On the other hand, our personal experience in our country is that many local health personnel are either unaware of this technique or underestimate its potential in local evaluations. Use of LQT could be expanded to assessment of a variety of other local health concerns, including infectious disease rates, prevalence of malnutrition, and completeness of household records, etc. Training of local health staff on how to use this technique in evaluating their local concerns could not only expedite the response to local needs but would also instill self-confidence in health personnel and motivate them to conduct small-scale research as well. Our study in Kecioren was an excellent example of this and stimulated other studies in the area.

In using LQT, it is important to recognize its assets and limitations and give them due consideration in planning local studies and assessing needs and potentials. Several biostatisticians and epidemiologists have long been working on validity of the LQT to decide whether it is an easy and quick yet "error-prone" method [9]. The LQT has been proven: 1) to have a high sensitivity (and high negative predictive value in most settings); thus, it is very good in detecting "low" performance if vaccine coverage is low, but 2) to have lower specificity and positive predictive value; thus, it may underestimate units' performance. LQT has been found very efficient and useful in situations where the population has an overall high coverage and sufficient performance, while subunits have heterogeneous performance levels, as in our example of Kecioren District. However, sample size calculations need to be done appropriately in order to reach valid estimates [10-13]. It is important to note that LQT does not claim to calculate exact coverage for individual lots.

In this study, we attempted to maximize the efficiency of the LQT by choosing *sample point areas*, from which children would be selected in a manner blinded to the children's living environment and/or distance to the health centers. Using local maps, each lot was divided into streets and 33 children were selected as scattered as possible, with preferably one child from each street, based on the total number of streets. Potential effects (if any) of "sharing a common environment" on vaccination status were thus minimized [14].

In vaccination-related studies, the source of information was found to be strongly associated with the validity and reliability of the information on vaccination status, site of vaccination, and reasons for non-vaccination [15]. Our study was based on self-reports, which could threaten the validity of our findings, leading to information bias. To minimize such an information bias, recall bias in particular, the "preferred" source of information in the study was the mother of the child. Additionally, in logistic modeling for identifying significant predictors of "full immunization", adjustment was made for the source of information, to control for its potential confounding effect.

The first aim of the study was to determine vaccine coverage in the District. Prevalence rate of 91.3% for full immunization in the Kecioren District was found to be far above the national average [16]. Despite small variations, coverage in the area is satisfactory for almost all vaccines, including BCG (99.4%), DPT-3 (98.5%), oral polio-3 (99.4%), and hepatitis B-3 (97.3%), and none of the "lots" had coverage of less than 75% for any of these vaccines. These rates point to the local health personnel's success in vaccination services. Interviewers also examined the BCG scar in 72% of the participants. Out of those checked for the BCG scar, 8.2% had no scar, although the child was reported by the parents as having a BCG vaccine. Given that parental self-report has the highest sensitivity value for BCG vaccine [15], this finding needs further attention. Future studies should check for BCG scars in individuals "validly" confirmed to be vaccinated with BCG, to determine whether our finding is a result of recall bias only or could suggest ineffectiveness of the BCG vaccination.

Despite the national campaign to eliminate measles [17], measles coverage among children in Kecioren District was found as 93.9% and fell below 75% in 5 lots (lots 10, 18, 21, 27, 33). In 2005, a LQT-based study was conducted in 40 cities in Turkey, and measles coverage was found as 94.1% in Ankara and 100% in Kecioren District [18]. One of the potential reasons for this conflicting situation could be the change in nationwide routine vaccination schedule against measles in 2006, before which the vaccine used was monovalent measles vaccine; it was later changed to trivalent MMR vaccine at the age of 12 months [18]. Our data collection period synchronized with this change in the national vaccination schedule. The change in vaccination schedule might have 1) caused a change in stocks or interruption in services in some regions, causing a decrease in coverage and/or 2) delayed the "acceptability" of services until parents learned about this new vaccine and the relevant schedule. Health personnel of the region eye-witnessed earlier that many families brought their children for vaccination at 9 months of age and were asked to return three months later, but did not return in the 12^th ^month for MMR. Among the major reasons for non-vaccination, many parents included in the study mentioned that "vaccination date was yet to come", although all children were at least 12 months old, probably because they had forgotten about the vaccination date for MMR. Of the 19 children who were not vaccinated against measles, with parents believing that they were young for the vaccine, 15 were < 15 months. In contrast with the national schedule, private physicians prefer to perform MMR at 15 months to maximize immunization rates, and this practice could have contributed to parents in the District being misinformed.

Before 2006, the usual explanation for a decrease in measles vaccination in routine surveillance reports was that "families take their children to private physicians and have MMR rather than monovalent measles vaccine". This can no longer be a valid explanation since the national program also includes MMR and our household survey would have identified such a possibility (if any), given that children were chosen regardless of their use of primary care services.

Finally, the data collection method could have caused erroneous results regarding coverage for measles vaccine. To eliminate such a possibility in the study, a child was treated as "vaccinated for measles" if the child was vaccinated with one dose of either monovalent measles or trivalent MMR. Of all children, 87.5% were vaccinated by measles and 14.4% were vaccinated by MMR, and 2% of children had one dose of MMR, following a monovalent measles vaccine at 9 months. Overall, 93.9% of children had at least one dose of measles vaccine. Another household survey may be conducted in Kecioren District next year to elucidate the potential reasons for a "low" coverage for measles and to guide further interventional activities (if needed).

Aside from estimation of coverage, the study aimed to examine various characteristics of children and their families, in an attempt to investigate how these factors might affect children's vaccination status. In parallel to the periodic reports of the District Health Administration, educational status of both mothers and fathers was above national averages. Young parental age (appropriate to having a 12–23-month-old child) and high in-migration rate of white-collar workers to this region (since the location is in a fast developing region in Ankara) can partially explain this relatively high educational level of the parents. Low occupational rates among these educated mothers could be explained by an interruption in employment or by the need for child care. The questionnaire had no inquiries regarding previous employment and/or any such interruption in occupational life; thus, this issue needs to be clarified in further studies.

In this study, two proxy measures were used to roughly estimate socioeconomic status and to investigate its potential effects on vaccination status. Almost all children had at least one parent working outside the home, earning an income. Family income needs to be interpreted carefully, however, given that information about salaries and expenses are missing. Of the children, only 14.3% had no health insurance. Taken together, these findings imply that area residents are "luckier" than their national counterparts in regards to their accessibility to health care services.

Occupational status of the parents was found to be associated with vaccination status, in that "full immunization" status was obtained in 94%, 91.4%, and 79.8% of children when both parents were employed, at least one parent was employed, or neither parent was employed, respectively (p < 0.001). This association stayed significant in logistic models, controlling for other factors: If either parent was working outside the home and earning an income, the child was 5.3 times more likely to be "fully immunized" than in cases in which both mother and father were unemployed, suggesting that employment could directly affect availability of and accessibility to vaccination services. To support this hypothesis, another related factor, presence of health insurance, was investigated to determine its association with "full immunization" status, but was found to be insignificant (p = 0.06). These conflicting results could be explained by the fact that vaccination services are provided free of charge in Turkey, and thus socioeconomic status and/or presence of health insurance do not directly affect availability and accessibility of services. Occupational status, on the other hand, may act as a "secondary factor" in leading to higher vaccination coverage, via its association with the parents' cultural status, social and mental health, inter-personal relationships, and their level of care and attention towards their children, etc.

Transmission of vaccine-preventable diseases to 12–23-month-old children may increase in the presence of any young sibling or household member, especially if s/he attends school, and also in crowded households. In the study group, 67% had at least one household member younger than 15 years. More than 60% had at least 1 and 5.3% had 3 or more siblings, and 42% of children had a household size of 5 or more individuals. These findings suggested a potential for high transmission rates for any contagious disease from family members, and with the finding of 16% of children living in slum settings, the risk for any oral-fecal or droplet infection in the household could be fairly high; thus, complete vaccination in the District is very important.

The prevalence of "full vaccination" was higher in families with 2 or more children (92%) compared to those with 1 child (90.4%) (p = 0.002), confirming the expectation that parental awareness of the importance of vaccines in childhood increases as the number of children in the family increases. Regardless of the number of children in the family, immunization status improves as the child ages (OR = 1.20). This latter situation implies that a major obstacle in the vaccination schedule in the District is a "delay", rather than a pause, as suggested by the reported reasons for non-vaccination (unavailability of the parents to take their child for vaccination on time, illness of the child at the time of vaccination, etc.).

In our study group, the vaccination site was mainly the health centers (82%), and this situation remained unchanged even in the service areas of the 3 MCH-FP centers, whose residents should be vaccinated by the MCH-FP center serving them. This finding could "truly" reflect that the health centers are the most preferred sites for vaccination, or it is possible that parents were unable to distinguish between a health center and MCH-FP center [19-21]. Either situation is a positive finding from a public health point of view, and indicates that vaccination services are mainly received from primary health care settings. Also encouraging is that accessibility to vaccination services does not seem to be a hindering factor against immunization, given that 60% of the children could reach vaccination services in less than 10 minutes.

One of the major criteria for "quality" of vaccination services is "owning a vaccination card". Vaccination cards inform both the parents and health personnel of the vaccination status of the child and timeliness and periodicity of vaccinations, alerts to any interruption in services, and reminds parents of the date of the "next" vaccination. In the study group, only 1.0% of the children reportedly had no vaccination card. In contrast, 41.3% of those with a card could not present the card to the interviewer upon request. It is not unexpected, though, and was also found earlier in an Indian study group, that more than half of the mothers do not keep vaccination cards for their children [15]. Health personnel working in the field should emphasize the importance of owning and retaining a vaccination card during their training and consultation services in the field and should motivate parents in this respect.

"Owning a vaccination card" is considered a quality measure in vaccination services. In the Kecioren District and in Turkey nationwide, health personnel are trained to provide a vaccination card for each child at his/her first visit for vaccination and to use this card on follow-up visits, for filling in the type and dose of each vaccine applied, along with the date of vaccination. In this study, parents were asked whether their children had any vaccination card and (if so) to show it to the interviewer at the time of the interview. The finding that prevalence of "fully immunized" children was significantly lower in the group with vaccination card who were able to show it upon request than in children without a card was an unexpected finding at first glance. It is unlikely that having an immunization card would make children less likely to be vaccinated; if anything, it would make children with cards more likely, because having a card and the ability to show it on request is probably an indicator of familial concern about immunization.

This finding could be the result of "recall bias". Families may misjudge their children's vaccination status and parents/caregivers may report that "all vaccines were done" even if a few doses of a vaccine are missing. Earlier studies on recall bias in vaccine-related research revealed that the type of vaccine affects parents' recall of their children's vaccination status. In a methodologic study involving 774 Indian children, one-year recall of mothers on immunization status of their children had a sensitivity value ranging between 53% (for measles) and 93% (for BCG), whereas specificity also widely varied, from 30% (BCG) to 68% (measles) [15].

Another potential source of error in the study could be a "social desirability bias", i.e., parents who only have to give their word, instead of providing a proof, could be more likely to claim that their children were immunized and mislead the interviewer, even when the child had missing doses/vaccines.

The cross-sectional and self-report-based nature of our study hinders our ability to further investigate why a negative association was identified between owning a vaccination card and full-immunization status. A social desirability bias seems to be the best explanation for our findings; such a bias does not invalidate the results, yet warrants further investigation in future studies. In vaccine-related studies, efforts should be maximized to obtain all available written information from vaccination cards, child follow-up cards, patient charts, etc., to increase validity of self-reports and to minimize the potential for information bias.

Our study also investigated the reasons for non-vaccination. Reasons did not vary significantly by the vaccine type (except for "young age" for measles, as discussed above) and seemed to be related with educational constraints rather than problems of availability, accessibility or acceptability. One frequently cited reason was "the child was sick and was not taken to the health care unit", which corresponds well to previous findings in local and national studies [18,19]. Families may hesitate to take their children for vaccination even when the child has a mild infection. It is important to educate parents regarding "valid" reasons for non-vaccination; the use of mass media in such activities will definitely increase efficiency of the activities. Many of the reasons reported in the study for non-vaccination, such as, "being unaware of a need for vaccination", "not knowing that a subsequent dose is also needed", "being away from home/area at the time for vaccination" and/or "familial reasons" were also suggested in a number of earlier studies conducted in Turkey [20,22,23]. Similarly, an important proportion of parents did not report a specific reason for non-vaccination, limiting the evidence to offer effective interventions. Future qualitative research (focus group discussions, in-depth interviews, etc.) on reasons for non-vaccination may be important and effective in this regard.

Lastly, the study pointed to the importance of emphasizing the vaccination date in educating/consulting parents on vaccine-related issues. Based on the nationally recommended schedule, "timeliness of vaccination" ranged between 63% and 87% in the study population. This calculation was based on data from those with a vaccination card and could represent those children only. Timeliness of vaccination could not be evaluated for those without a vaccination card, while those with a card but who could not show it at the time of the interview would have similar, if not lower, prevalence rates.

In summary, as reasons associated with vaccination failure may vary in each nation, region, and/or time period, it is important to evaluate coverage periodically and to determine reasons for non-vaccination (if any) to plan interventional activities customized specifically in regards to the associated status and needs, with an ultimate goal of maximizing childhood vaccine coverage nationwide. In consideration of the results of such a study in Kecioren District, the relatively high educational and socioeconomic status of parents alone could not minimize the "considerable" risk of vaccine-preventable diseases in the District and commands continuity of efforts for improving vaccination rates. We believe the high rate of non-vaccination against measles should be further investigated in future case-based surveillance and/or coverage studies; administrative methods should be backed up by household surveys to strengthen vaccination monitoring in the District; and families should be trained and motivated to have their children's vaccines completed in full and in a timely manner according to the recommended schedule.

## Conclusion

The Lot Quality Technique, years after its introduction to health-related research, is still an appealing technique for rapid evaluation of the extent of a variety of local health concerns in developing countries, and in rural areas in particular, and is very efficient in determining performance of individual subunits in a given service area. Thus, training of local health personnel on use of LQT could expedite response to local health problems and would even motivate them in conducting their own surveys tailored to their professional interests.

## Competing interests

The authors declare that they have no competing interests.

## Authors' contributions

BC designed and developed the research study, analyzed the data and wrote the manuscript. SU developed the research study, managed the research and edited the manuscript. FT developed the study, input and analyzed the data and assisted in writing the manuscript. LA developed the study and edited the manuscript. All authors have read and approved the final manuscript.

## Pre-publication history

The pre-publication history for this paper can be accessed here:


